# Genome-Wide Association of Histone H3 Lysine Nine Methylation with CHG DNA Methylation in *Arabidopsis thaliana*

**DOI:** 10.1371/journal.pone.0003156

**Published:** 2008-09-08

**Authors:** Yana V. Bernatavichute, Xiaoyu Zhang, Shawn Cokus, Matteo Pellegrini, Steven E. Jacobsen

**Affiliations:** 1 Molecular Biology Institute, University of California Los Angeles, Los Angeles, California, United States of America; 2 Department of Molecular, Cell and Developmental Biology, University of California Los Angeles, Los Angeles, California, United States of America; 3 Department of Plant Biology, University of Georgia, Athens, Georgia, United States of America; 4 Howard Hughes Medical Institute, University of California Los Angeles, Los Angeles, California, United States of America; University of California Davis, United States of America

## Abstract

Methylation of histone H3 lysine 9 (H3K9) is a hallmark of transcriptional silencing in many organisms. In *Arabidopsis thaliana*, dimethylation of H3K9 (H3K9m2) is important in the silencing of transposons and in the control of DNA methylation. We constructed a high-resolution genome-wide map of H3K9m2 methylation by using chromatin immunoprecipitation coupled with whole genome Roche Nimblegen microarrays (ChIP-chip). We observed a very high coincidence between H3K9m2 and CHG methylation (where H is either A,T or C) throughout the genome. The coding regions of genes that are associated exclusively with methylation in a CG context did not contain H3K9m2. In addition, we observed two distinct patterns of H3K9m2. Transposons and other repeat elements present in the euchromatic arms contained small islands of H3K9m2 present at relatively low levels. In contrast, pericentromeric/centromeric regions of Arabidopsis chromosomes contained long, rarely interrupted blocks of H3K9m2 present at much higher average levels than seen in the chromosome arms. These results suggest a complex interplay between H3K9m2 and different types of DNA methylation and suggest that distinct mechanisms control H3K9m2 in different compartments of the genome.

## Introduction

Multiple mechanisms control the formation and maintenance of eukaryotic heterochromatin. One important conserved mechanism is the presence of particular post-translational modifications of histones, such as methylation. Histones can be mono, di or tri methylated (m, m2, or m3) and each methylation state can be controlled by different histone methyltransferase enzymes and can be associated with different outcomes [1]. For example, mammalian histones modified with tri-methylation at lysine 9 of histone H3 (H3K9m3) are found in regions of silenced chromatin [2], [3]. H3K9m3 is associated with pericentromeric heterochromatin and crucial for proper mammalian development, as mutants in H3K9m3 histone methyltransferases, Suv39h1 and Suv39h2, display genomic instability and impaired viability [3]–[5]. In Arabidopsis, di-methylation of histone H3 lysine 9 (H3K9m2) is associated with heterochromatin formation, while H3K9m3 is found throughout euchromatin [6]. Several histone methyltransferases are responsible for the propagation of H3K9m2, including KRYPTONITE/SUVH4 (KYP), SUVH5 and SUVH6 [7]–[10]. In addition to eliminating H3K9m2, *kyp suvh5 suvh6* triple mutants also reduce DNA methylation and lead to the loss of silent epigenetic states of heterochromatin as observed by transposon reactivation [9].

DNA methylation in Arabidopsis is present in three DNA sequence contexts: CG, CHH and CHG (where H = A, T, or C). The initial establishment of methylation (*de novo* methylation) in all three sequence contexts requires the *DOMAINS REARRANGED METHYLASE 2* DNA methyltransferase (DRM2) [11]. DRM2 appears to be guided by 24 nucleotide small interfering RNAs (siRNAs), because the establishment of methylation is blocked by mutations in several RNA silencing genes that control siRNA biogenesis or utilization including *ARGONAUTE4 (AGO4)*, *RNA DEPENDENT RNA POLYMERASE2 (RDR2)*, *DICER-LIKE3 (DCL3)*, *RNA POLYMERASE IVa (NRPD1a)*, *RNA POLYMERASE IVb (NRPD1b)*, and *DRD1* [12]–[19]. The mechanisms involved in the maintenance of DNA methylation are different depending on the sequence context of the cytosine [20], [21]. CG methylation is maintained by the DNA methyltransferase MET1, which is a homolog of mammalian Dnmt1 [20], [22]–[27], and by an accessory factor called UHRF1 in mammals or VIM/ORTH in Arabidopsis [28]–[31]. CHH methylation is maintained by mechanisms very similar to that of *de novo* methylation, because *drm2* and the same suite of RNA silencing mutants cause losses of CHH methylation [20]. However, the DNA methyltransferase CHROMOMETHYLASE3 (CMT3) also plays some role in CHH maintenance and acts redundantly with DRM2 at some loci [11]. CMT3 is also the main enzyme acting to maintain CHG methylation throughout the genome, however, at some loci DRM2 also plays an important role [11], [32].

CHG DNA methylation was found to be linked to H3K9m2 when screens for mutations that reduce CHG DNA methylation uncovered mutations in the *KYP* locus encoding a histone H3 methyltransferase [7], [33]. *kyp* mutations were shown to specifically reduce H3K9m2 *in vivo*, and KYP is efficient at mono- and di- methylation, but not trimethylation, of H3K9 sites *in vitro* [34]. The mechanism by which H3K9m2 controls CHG DNA methylation is not yet clear but appears to involve direct recruitment of CMT3 to methylated histones because CMT3 contains a chromodomain that is capable of binding directly to methylated histone peptides [35]. Efficient binding of CMT3 chromodomain to methylated histones *in vitro* required simultaneous methylation of both the lysine 9 and lysine 27 positions, suggesting that H3K27 methylation may also be involved in the recruitment of CMT3 [35]. More recently, the SRA domain present within KYP was shown to bind *in vitro* to oligonucleotides that are methylated at CHG sites, suggesting that KYP is directly recruited to methylated DNA [30]. These results suggest a self-reinforcing feedback loop between CMT3 and KYP that would ensure efficient maintenance of CHG DNA methylation.

Recent genome-wide profiling studies of DNA methylation within the Arabidopsis genome utilizing either microarrays or whole genome shotgun bisulfite sequencing have revealed key aspects of DNA methylation patterning [32], [36]–[40]. CG, CHG and CHH methylation are highly correlated with each other and with transposons and other repeat sequences throughout the genome. An interesting exception, however, is in the coding region of genes (gene bodies), where only CG methylation is found [32], [39], [40]. Gene body methylation occurs on about a third of all genes, and these genes tend to be highly and ubiquitously expressed in different Arabidopsis tissues [39], [40]. The function of this methylation is unclear, but it has been proposed to be involved in the suppression of cryptic transcription initiation which would otherwise interfere with transcription from the 5′ promoter [36], [40].

Multiple studies have also addressed the location of H3K9m2 either by utilizing chromatin immunoprecipitation (ChIP) at individual loci, or by using a ChIP-microarray (ChIP-chip) approach with a microarray consisting of 1-kb probes tiled across Arabidopsis chromosome 4 [41]–[45]. These studies indicated that H3K9m2 is highly enriched on transposons and pseudogenes and is also associated with repeat elements. In order to gain deeper insight into H3K9m2 methylation patterning, we profiled H3K9m2 by using native nucleosome chromatin immunoprecipitation (native ChIP), coupled with a high resolution Nimblegen array containing ∼60 nucleotide probes tiled across the entire Arabidopsis genome and combined these data with recently published DNA methylation profiling data. We found that H3K9m2 was specifically associated with CHG DNA methylation throughout the genome, and not with body methylated genes that only contain CG methylation. Furthermore, we found that pericentromeric heterochromatin and euchromatic chromosome arms show distinct patterns of H3K9m2, suggesting that different targeting mechanisms exist for these different genome compartments.

## Results and Discussion

### Genome-wide detection of H3K9m2

We designed a Roche Nimblegen High Density (HD2) tiling array with 1.98 million probes spaced every 60 nucleotides and covering the entire sequenced portion of the Arabidopsis genome. By tiling across the genome, all repeated sequences were included and were represented proportionally to their copy number. We utilized a native chromatin immunoprecipitation (ChIP) method in which the crosslinking/sonication step was eliminated to achieve nucleosome resolution data. Chromatin was subjected to limited digestion with Micrococcal nuclease (MNase) such that the majority of the chromatin was present as mononucleosomes (Figure S1a). Digested chromatin was then immunoprecipitated with a highly specific monoclonal antibody against di-methylated lysine 9 on histone H3 (H3K9m2) (Figure S1b). ChIP with antibody against the unmodified C-terminus of histone H3 was used as a control [46]. DNA fragments extracted from either the H3K9m2 or control immunoprecipitation were amplified, labeled with either cy3 or cy5, and hybridized to the tiling arrays. Three biological replicates experiments were performed and each probe was assigned a Z-score as detailed in Material and Methods. Based on the distribution of Z-scores, we defined a positive probe as having a score higher than 0.2 (Figure S2). In order to minimize false positive signals, we defined a set of “methylated regions” utilizing max gap/min run settings that allow detection of regions not shorter than the size of a mononucleosome (147 bp) (Material and Methods).

The level of false positive signals was evaluated by including probes on the array corresponding to the entire chloroplast genome (154,478 bp sequence), which lacks histones and therefore H3K9m2. Only 8 regions (1.19%) were falsely identified as being H3K9m2 positive. Comparing this false positive rate to the 22.5% positive probes for the nuclear genome, suggests that the majority of signals in the nuclear genome represent true signal. The reproducibility of the microarray data was assessed in two ways. First, we performed additional Nimblegen profiling experiments utilizing a conventional, formaldehyde crosslinking ChIP method [46]. The correlation between the two sets of data was very high (Pearson correlation coefficient = 0.9) (Figure S3). Second, we compared our results with a list of H3K9m2-positive regions compiled from previously published studies (Table S1). We confirmed H3K9m2 methylation of 99.6% of previously published regions [41]. These results suggest that the experimental method and the microarray platform allow for efficient detection of H3K9m2.

### A high correlation between H3K9m2 and CHG DNA methylation

**Figure 1:**
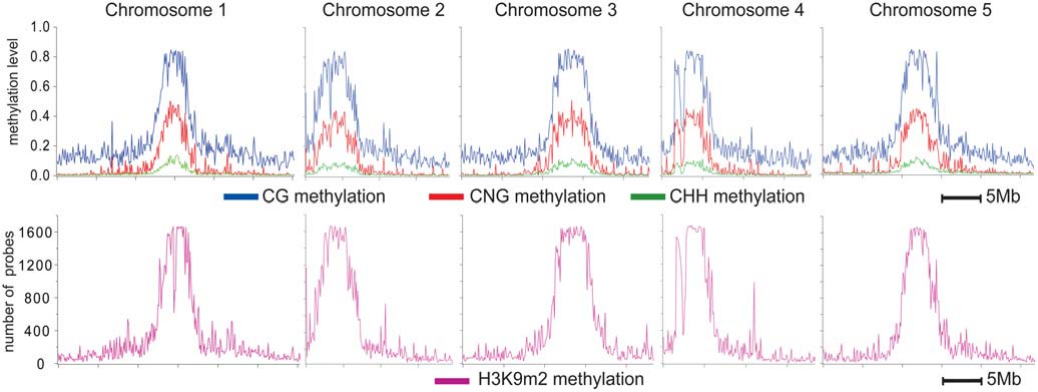
Distribution of DNA methylation and K9H3m2 methylation along chromosomes. Top panels show average DNA methylation levels in a sliding 100 kb window [32]. Bottom panels show the number of H3K9m2 positive probes (z-score>0.2) in a sliding 100 kb window.

We identified 17,099 regions with significant levels of H3K9m2, covering a total of 27 Mb of the sequenced genome (22.5%). Figure 1 shows a distribution of H3K9m2 along each of the five Arabidopsis chromosomes in sliding 100 bp window. Consistent with previous reports and immunofluorescence studies [10], [41], [47], H3K9m2 is highly enriched in Arabidopsis pericentromeric regions and is correlated with DNA methylation (Figure 1).

**Figure 2:**
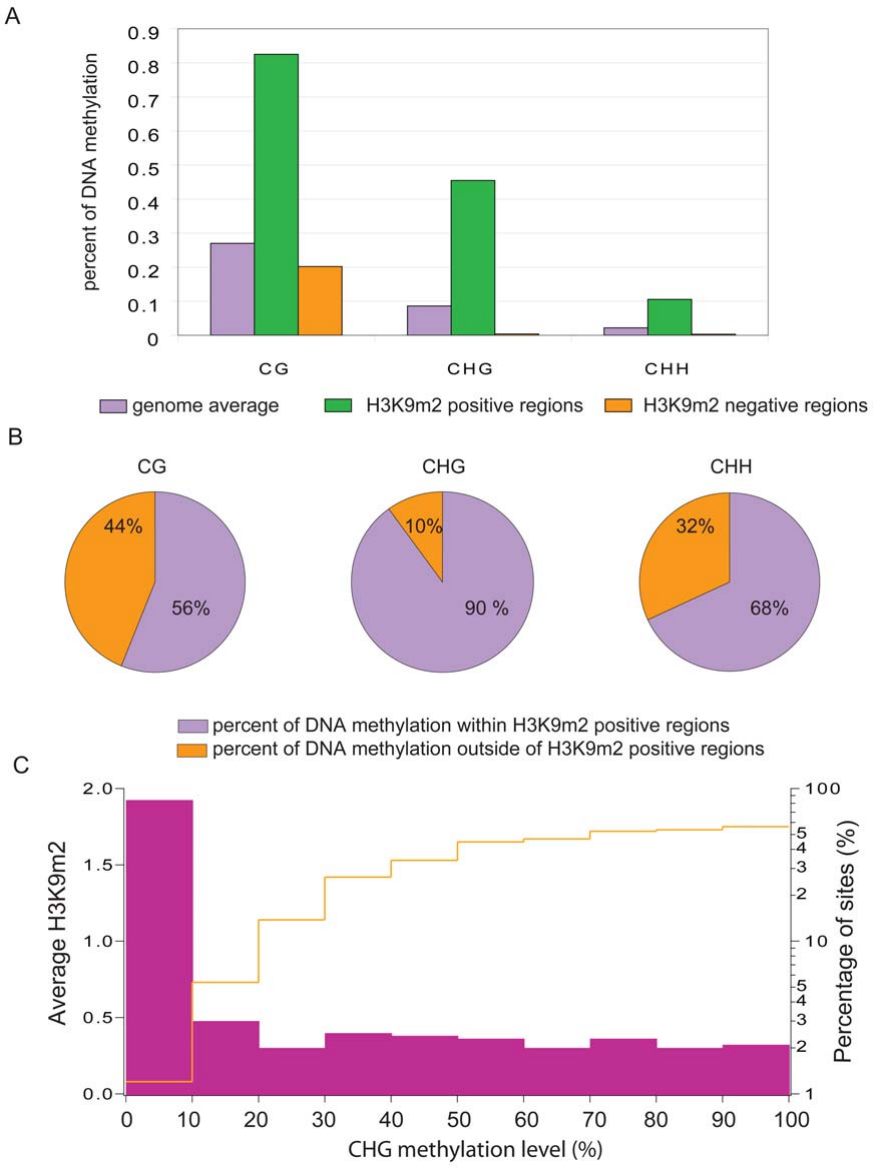
Correlation of H3K9m2 methylation with CHG DNA methylation. (a) Average DNA methylation levels genome wide (purple), in H3K9m2 positive regions (Z score>1, green) and in H3K9m2 negative regions (Z-score<0, orange). (b) Percent of CG, CHG and CHH methylation falling within H3K9m2 positive regions. (c) Correlation of the percentage of CHG methylation at individual sites throughout the genome with H3K9m2 levels. The x-axis is divided onto 10 individual bins that represent deciles of the percentage of CHG methylation of all CHG sites in the genome. Purple bars represent the percentage of CHG sites that fall with each methylation decile (right logarithmic axis), and orange line represents the average H3K9m2 level (Z-score) (left axis).

In order to correlate H3K9m2 with DNA methylation in the different sequence contexts, CG, CHG and CHH, we compared the H3K9m2 profiling data with whole genome shotgun bisulfite sequencing (BS-seq) data, which represents quantitative single nucleotide resolution data for individual cytosines throughout the genome [32]. First, we calculated average DNA methylation percentages within both H3K9m2 highly positive and negative probes (with Z-scores higher than one and lower than zero, respectively) (Figure 2a). All three kinds of DNA methylation were highly enriched within H3K9m2 positive probes, compared to genome average levels. However, H3K9m2 negative probes were virtually devoid of CHG and CHH methylation, while CG methylation was still present. Second, we asked what fraction of methylated cytosines (defined with the thresholds of >50% for CG, >20% for CHG and >0% for CHH) fall within H3K9m2 positive regions (as defined as regions with Z-scores>0.2). Figure 2b shows that only a very small fraction of CHG methylation does not overlap with these H3K9m2 positive regions (also see examples in Figure S4), while CG and CHH methylation shows a higher level of non-overlap. The relatively high fraction of CG methylated regions that do not overlap with H3K9m2 positive regions corresponds to genes (Figure S5), consistent with previous observations of CG-only methylation in the transcribed region (body) of genes. Thus, H3K9m2 is tightly linked to CHG methylation throughout the genome, and is not present at CG-only methylated genes.

CHG methylation at individual sites shows a broad distribution of methylation levels from 20% to 100%. To test if H3K9m2 levels correlate with different CHG methylation levels, we computed the average H3K9m2 level within each decile of CHG methylation level (Figure 2c). Regions with CHG methylation lower than 10% were mostly devoid of H3K9m2. In contrast, CHG sites methylated at levels between 10% and 50% showed a monotonically increasing level of H3K9m2. This quantitative correlation between H3K9m2 and CHG DNA methylation is consistent with the molecular genetic characterization of KYP and CMT3 [7], [30], [33]–[35] and suggests that the linkage between CHG methylation and H3K9m2 is a genome-wide phenomenon. Interestingly, CHG sites methylated at a level between 50% and 100% showed a relatively uniform level of H3K9m2, possibly because this is the maximum level of H3K9m2 attainable.

### Distinct patterns of H3K9m2 in pericentromeric heterochromatin and euchromatic arms

**Figure 3:**
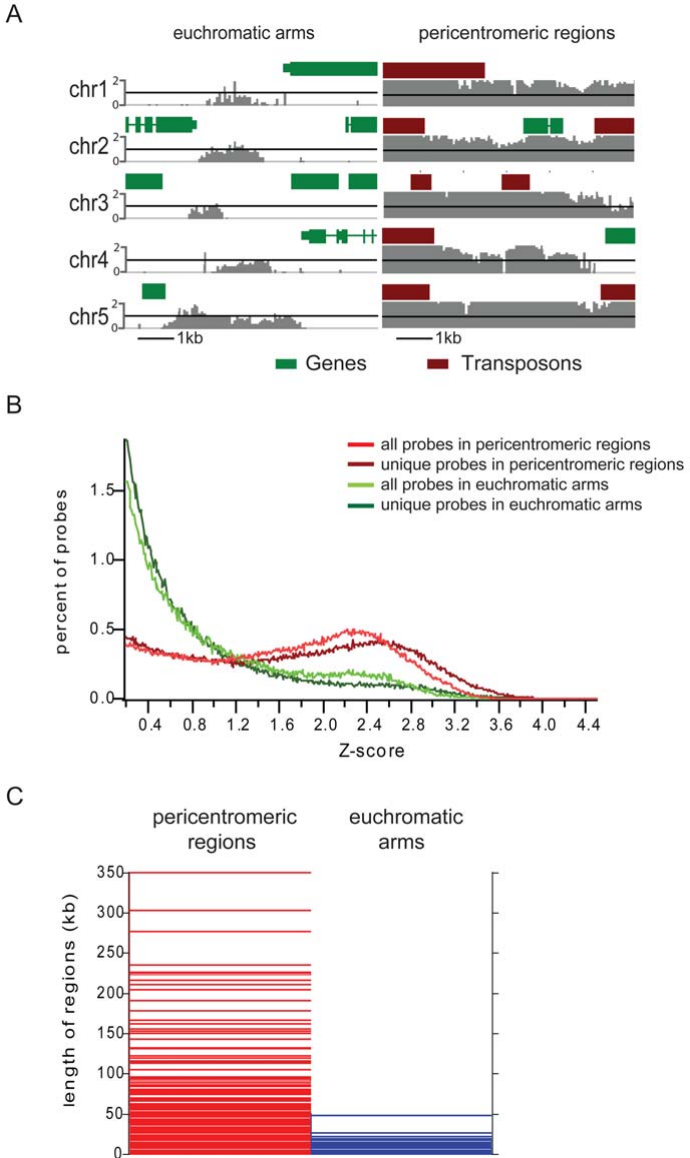
Relationship between H3K9m2 levels and the length of H3K9m2 positive regions in different chromosomal compartments. (a) Examples of typical H3K9m2 levels present in either euchromatic chromosomal arms or pericentromeric regions. Each gray bar corresponds to the Z-score of an individual probe. (b) Distribution of z-scores in the euchromatic arms (green) and pericentromeric regions (red). (c) Lengths of uninterrupted H3K9m2 methylated regions, with each horizontal bar representing a single region in either the euchromatic arms or pericentromeric regions.

Inspection of the H3K9m2 profiling data in the UCSC genome browser revealed dramatic differences in the patterns of H3K9m2 in pericentromeric heterochromatin and the euchromatic arms (Figure 3a). Pericentromeric regions contained long, uninterrupted regions of high levels of H3K9m2, while euchromatic arms showed smaller, isolated patches of H3K9m2 with overall lower levels. To quantitate this phenomenon, we assigned regions of the five Arabidopsis chromosomes to either pericentromeric regions or regions corresponding to the euchromatic arms of the chromosomes, based on the overall abundance of repeats, genes and DNA methylation (Figure S6), and then analyzed these two genome compartments separately. We observed that overall H3K9m2 levels, as measured by the distribution of Z-scores of individual probes, were higher in pericentromeric regions (median score of 1.78) than in the arms (median score of 0.74). The majority of H3K9m2 positive probes (those with Z-scores>0.2) in chromosome arms had a Z-score lower than 1.2, while the majority of probes in pericentromeric regions had scores higher than 1.2 (Figure 3b). To avoid the potential ambiguity introduced by cross-hybridization of repetitive sequences, we performed the same test using only probes with unique sequences (probes that match only once to the genome as defined by BLAT analysis) [48]. Consistent with the observations for all probes, the distribution of Z-scores in chromosome arms was generally lower than the distribution in pericentromeric regions (Figure 3b). To eliminate the possibility that this difference is caused by insensitivity of pericentromeric regions to MNase digestion, we made similar comparisons utilizing data obtained from formaldehyde crosslinking ChIP experiments (Figure S7). We observed the same general trend in this dataset, with a higher proportion of pericentromeric probes showing high Z-scores. Another distinction between H3K9m2 patterns in pericentromeric regions and chromosome arms is the length of H3K9m2 positive regions (Figure 3a and 3c). H3K9m2 in pericentromeric regions tended to occupy large domains, showing an average length of 3.4 kilobases and a maximum length of 350 kilobases (Figure 3c). Conversely, the average length of H3K9m2 positive regions in chromosome arms was only 0.6 kilobases.

**Figure 4:**
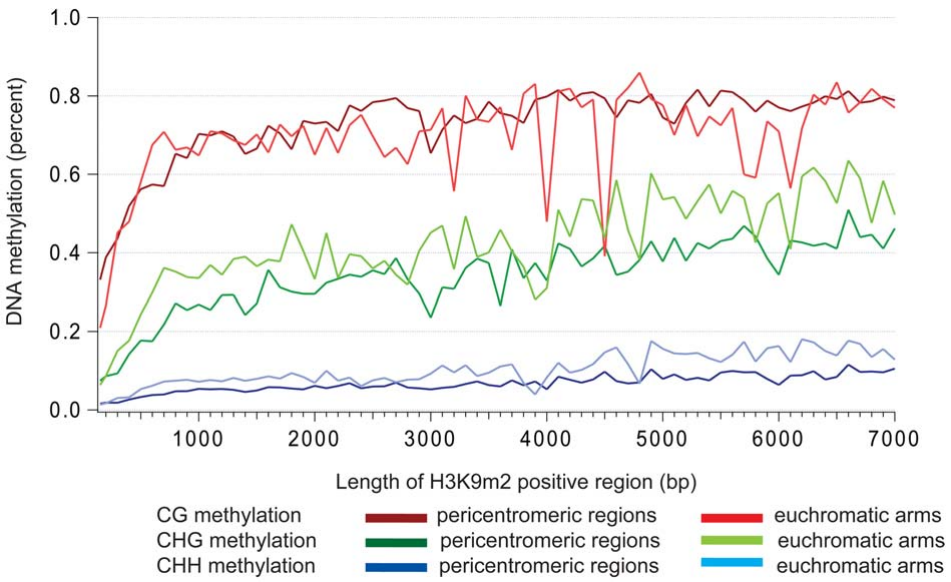
The percentage of DNA methylation increases as the length of H3K9m2 positive regions increases. Average percent DNA methylation of CG (red), CHG (green) and CHH (blue) was calculated for H3K9m2 positive regions of different lengths.

Because of the strong correlation between CHG methylation and H3K9m2, and because H3K9m2 levels in pericentromeric heterochromatin are generally higher than in the euchromatic arms, we expected that CHG methylation would also be higher in pericentromeric regions than in the arms. Surprisingly however, when we analyzed the average CHG levels from whole genome shotgun sequencing data [32], we found that H3K9m2 positive regions in pericentromeric heterochromatin showed lower overall levels of CHG methylation (19.9%) than in H3K9m2 positive regions in the arms (35.9%). We also analyzed the relationship between CHG methylation levels and the length of the H3K9m2 positive regions in either pericentromeric heterochromatin or the euchromatic arms. As previously seen for specific elements of the genome [32], we found a positive correlation between the length of a region and the percentage of CHG methylation (Figure 4). This analysis again showed that at any given length of H3K9m2-positive region, average levels CHG methylation are lower in pericentromeric heterochromatin than in the arms.

Our results indicate that H3K9m2 tends to be distributed in large blocks in pericentromeric regions, while in chromosome arms H3K9m2 forms smaller regions that have lower levels of H3K9m2 with higher levels of CHG methylation. While it is not clear why these differences exist, the data suggest that H3K9m2 may be regulated differently in these two compartments of the Arabidopsis genome. One possibility is that pericentromeric heterochromatin may utilize a mechanism not used in chromosome arms that allows for the spreading of H3K9m2 to fill large domains and to target higher levels of H3K9m2. For instance, it has been proposed that pericentromeric heterochromatin may more efficiently replicate epigenetic states during chromatin replication, whereas smaller elements in the chromosome arms may require more active targeting by siRNA mediated processes because they are too small for efficient transmission of epigenetic information between adjacent nucleosomes [38]. This more active targeting may also explain the higher levels of CHG DNA methylation seen in euchromatic arms. Regardless of the mechanism, these findings underscore a possible difference in the targeting or maintenance of H3K9m2 in different genome compartments.

### Association of H3K9m2 with genes

**Figure 5:**
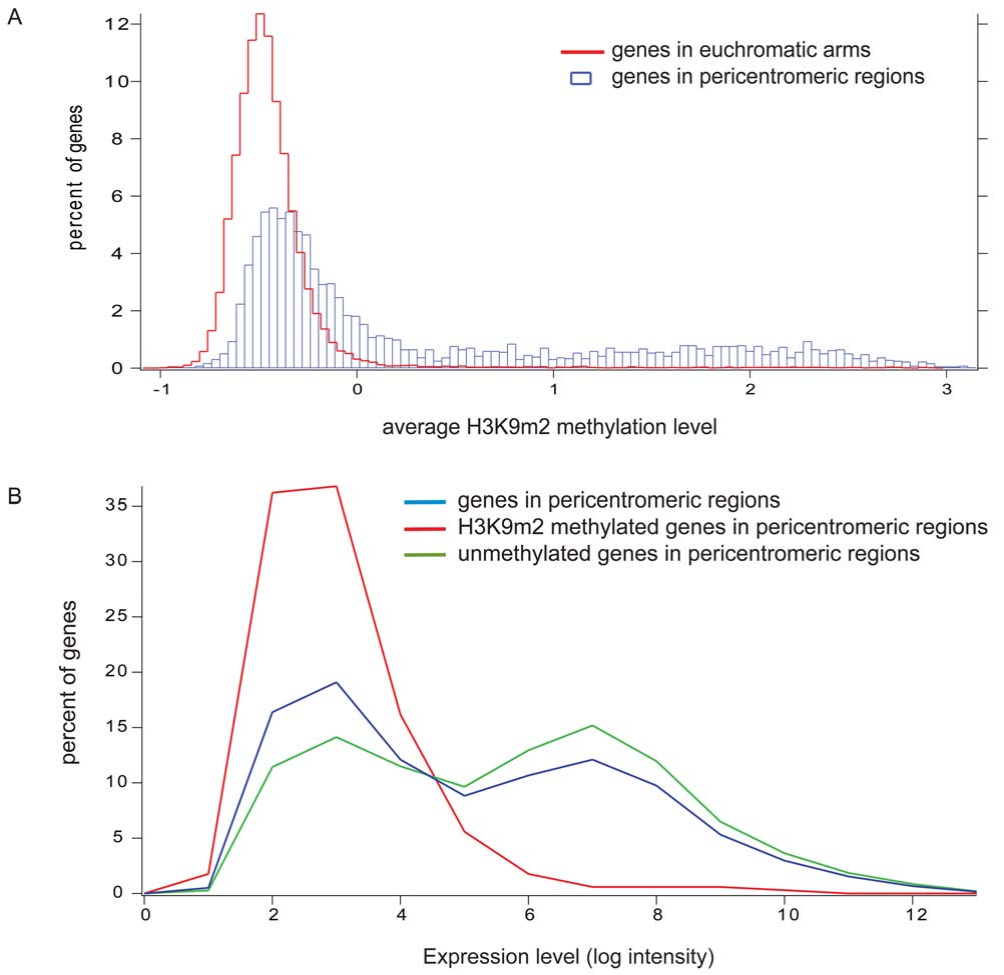
H3K9m2 methylation and expression levels of genes. (a) Histogram of H3K9m2 methylation of genes present in euchromatic arms (red line) or present in pericentromeric regions (blue bars). X-axis corresponds to average Z-score of each gene and Y-axis is the percent of the genes falling within each Z-score interval. (b) Expression level of all genes located in pericentromeric regions (green), those that are H3K9m2 methylated (red) or are unmethylated (blue).

We analyzed the distribution of average levels of H3K9m2 within protein coding genes found in the euchromatic arms of chromosomes and found that H3K9m2 levels are very low in the vast majority of genes (Figure 5a). These results are consistent with the nature of H3K9m2 as a gene silencing mark, and with whole genome bisulfite sequencing results showing that the majority of genes are devoid of DNA methylation in their promoters, and lacking in CHG and CHH methylation in their coding regions [32], [37]. It is also consistent with our finding that H3K9m2 is not associated with CG-only gene body DNA methylation (Figure S4).

We also analyzed levels of H3K9m2 within the 3518 genes present in pericentromeric heterochromatin and found that 31% had significant levels of H3K9m2 methylation (average score is higher than 0.5), while the majority of genes (75%) were devoid of H3K9m2 (Figure 5a). These results are consistent with earlier findings that genes present in heterochromatic regions “escape” targeting by H3K9m2 and DNA methylation and thereby avoid gene silencing [47], but also show that a significant minority of the genes are indeed associated with H3K9m2.

We utilized publicly available microarray expression data averaged over 79 tissues and conditions [49] to analyze expression levels of 2049 of the 3518 pericentromeric genes which were present on the ATH1 array used for expression analysis. We found that the 547 pericentromeric genes with high H3K9m2 levels (27%) were expressed at very low levels compared to the unmethylated pericentromeric genes (Figure 5b). In contrast, genes lacking in H3K9m2 showed a distribution of expression levels (Figure 5b) similar to those previously reported for all protein coding genes in the genome (see Figure 3 in [39]). These results are consistent with previous observations that H3K9m2 methylation is usually associated with gene silencing and show that genes that escape H3K9m2 silencing in pericentromeric regions show normal levels of expression.

### Association of H3K9m2 with transposons, repeat elements and endogenous siRNAs

**Figure 6:**
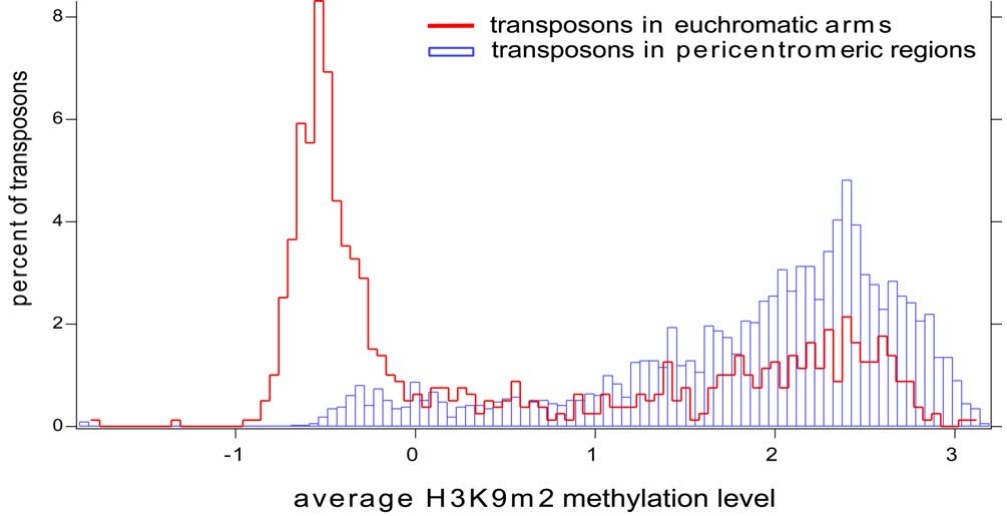
H3K9m2 methylation of transposable elements. The majority of transposons in pericentromeric regions have high levels of H3K9m2 (blue bars), while a large number of transposons in the arms are unmethylated (red line). X axis represents the average H3K9m2 level (Z-score) for the element and Y axis represents the percentage of elements falling into each category.

Transposons (which are often annotated as pseudogenes in the Arabidopsis genome) are frequently DNA methylated and silenced, and associated with H3K9m2 [7], [32], [33], [37], [39], [41]–[43], [47]. We examined H3K9m2 levels of transposons/pseudogenes located in pericentromeric regions (77% of all elements) and in the euchromatic arms (23%). Figure 6 shows that transposons/pseudogenes located in pericentromeric regions are much more frequently associated with high levels of H3K9m2 than are transposons/pseudogenes in the euchromatic arms.

**Figure 7:**
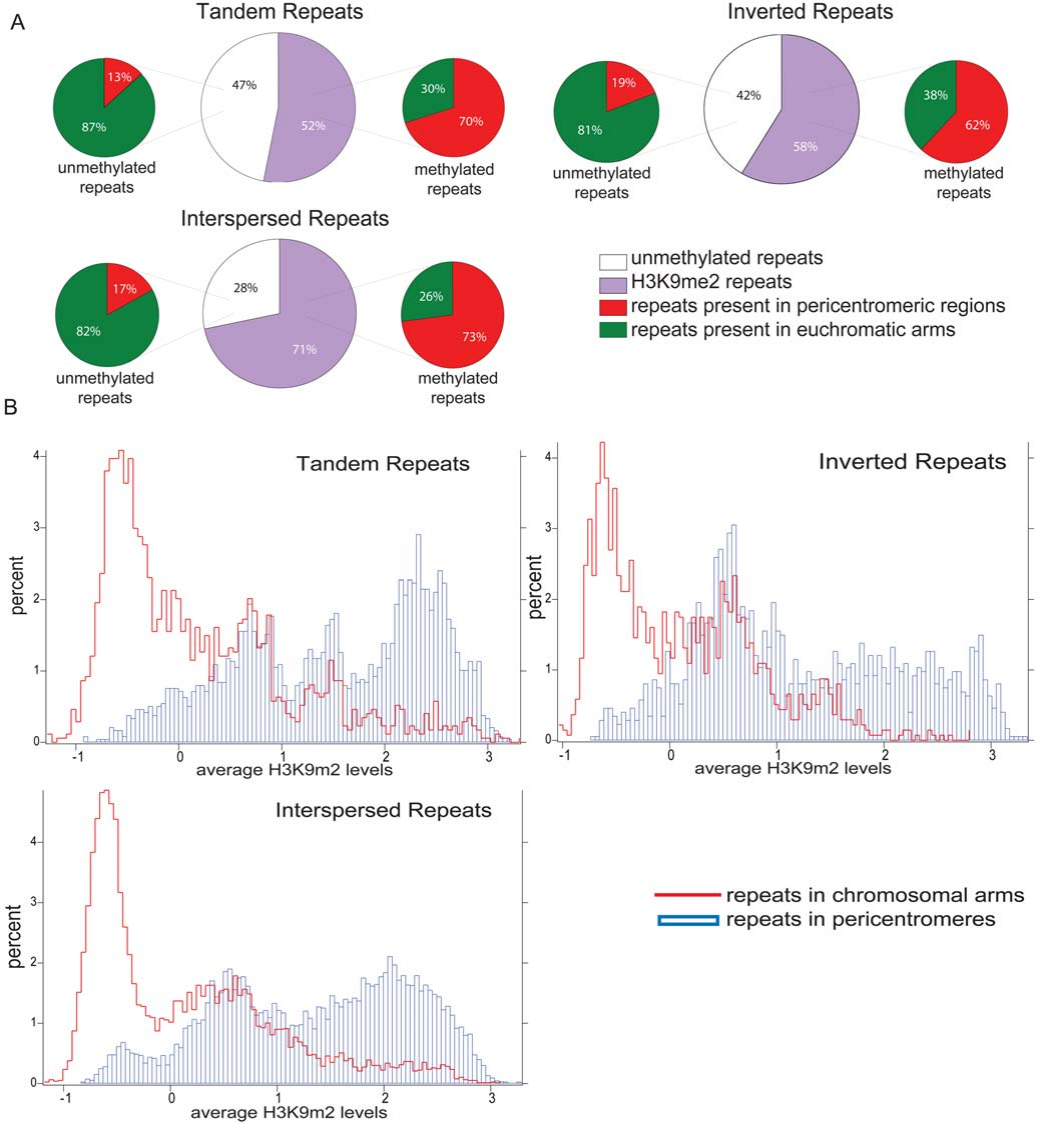
H3K9m2 of various repeat elements in the Arabidopsis genome. (a) Percent of H3K9m2 methylated (purple) and unmethylated repeats (white). The majority of unmethylated repeats are present in euchromatic arms (green), while the majority of methylated repeats are located in pericentromeric regions (red). (b) Average H3K9m2 levels across tandem, inverted and interspersed repeats present in different genome compartments. All three kinds of repeats are more frequently methylated and have higher average H3K9m2 levels when they reside in pericentromeric regions. X axis represents the average H3K9m2 level (Z-score) for the element and Y axis represents the percentage of elements falling into each category.

We also analyzed three classes of DNA repeats for H3K9m2 methylation (tandem repeats, inverted repeats, and interspersed repeats), and found a much higher frequency of H3K9m2 on the repeats present in pericentromeric regions as compared to those found in the euchromatic arms (Figure 7a). The majority of unmethylated repeats (more than 80%) were found in the euchromatic arms. The fact that many repeats remain unmethylated in the euchromatic arms (for instance 4420 out of 7778 tandem repeats and 2428 out of 3471 inverted repeats), suggests that repeat character alone may not be sufficient for recognition and silencing. These findings also underscore the unique nature of silencing in pericentromeric regions, which encompasses large swaths of silent chromatin and encompasses many repeat elements.

We examined the distribution of H3K9m2 levels of repeats found in either pericentromeric heterochromatin or euchromatic arms (Figure 7b). We found that a significant fraction of repeats found in pericentromeric heterochromatin showed a peak of very high H3K9m2 levels, which was higher than the levels found in H3K9 methylated repeats in the arms. These results are consistent with the general trend of very high levels of H3K9m2 in pericentromeric regions, and suggest that many types of repeated DNAs become highly H3K9 methylated in these regions.

**Figure 8:**
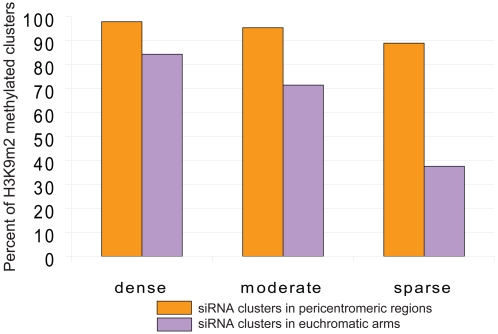
H3K9m2 methylation of siRNA clusters. Graph shows the percentage of different categories of siRNA clusters (dense, medium, or sparse) that overlap with H3K9m2 positive regions in either pericentromeric regions (orange) or arms (purple).

Abundant evidence shows that small interfering RNA (siRNAs) can recruit DNA methylation and gene silencing [50]. We carried out an analysis of H3K9m2 within clusters of endogenous siRNAs defined by massively parallel signature sequencing (MPSS) [51] by determining how many of those clusters fall within H3K9m2 regions. As expected, the majority of siRNA clusters present in pericentromeric regions were highly associated with H3K9m2 (90% or higher overlap), independent of whether the siRNA cluster was defined as dense, moderate or sparse (Figure 8). However, in the euchromatic arms of the chromosomes, sparse clusters behaved differently from the dense/moderate clusters. In particular, while the percent of H3K9m2 dense clusters stayed relatively high (84%), the percent of H3K9m2 methylated sparse clusters decreased to 37%. These results indicate that dense siRNA clusters are strongly associated with H3K9m2, independent of their position in the genome, while sparse siRNA clusters are not as efficient at recruiting H3K9m2 in euchromatic arms. These findings also once again underscore the differences between H3K9m2 patterns in pericentromeric heterochromatin relative to euchromatic arms.

### Conclusion

We have profiled H3K9m2 methylation across the Arabidopsis genome and combined the analysis with recently published high-resolution BS-seq data to understand the relationship between H3K9m2 and DNA methylation. The H3K9m2 methylation data, along with repeat annotations and other epigenomic datasets are available to the community at our local UCSC genome browser site: http://epigenomics.mcdb.ucla.edu/H3K9m2/

Our bioinformatics analyses, as well as manual inspection of the data in genome browsers, shows that H3K9m2 and CHG DNA methylation are very tightly correlated throughout the genome, showing that the relationship between these two marks may be universal. These results are consistent with the proposed self-reinforcing loop mechanism operating between the CHG methyltransferase (CMT3) and the primary H3K9m2 methyltransferase KYP [30]. Our data also show that the CG only methylation associated with roughly one third of expressed genes is not correlated with H3K9m2, showing that this type of methylation is maintained by a different mechanism. Finally, our data suggest that different mechanisms are operating to maintain high levels of H3K9m2 in large blocks of pericentromeric heterochromatin, and smaller patches of H3K9m2 on repeats and transposons found in the euchromatic arms of the chromosomes. Future studies will hopefully uncover the mechanistic differences between these two patterns of epigenetic silencing.

## Methods

### Native chromatin immunoprecipitation

The entire shoots of three week old Arabidopsis plants (Col-0 ecotype), grown under continuous light, were harvested, frozen in liquid nitrogen and ground to powder (1 g). Plant tissue was resuspended in 10 ml of HBM buffer (25 mM Tris-Cl pH 7.6, 440 mM sucrose, 10 mM MgCl_2_ and 0.1% Triton-X, 10 mM β-mercaptoethanol, 2 mM spermine, 1 mM PMSF, 1 ug/ml pepstatin and EDTA-free protease inhibitor cocktail (Roche), homogenized and filtered through Miracloth (Calbiochem). After spinning at 3000 rpm for 5 min (SS-34, Sorvall), the pellet was resuspended in 5 ml of NIB buffer (20 mM Tris-Cl pH 7.6, 250 mM sucrose, 5 mM MgCl_2_, 5 mM KCl, 0.1% Triton-X, 10 mM β-mercaptoethanol), applied to a 15/50% Percoll (GE Healthcare) gradient in NIB and spun 2000 rpm for 20 min (SS-34, Sorvall). Isolated nuclei were washed two times in NIB buffer and flash frozen in liquid nitrogen in HBC buffer (25 mM Tris-Cl pH 7.6, 25 mM Tris-Cl pH 7.6, 440 mM sucrose, 10 mM MgCl_2_ and 0.1% Triton-X, 10 mM β-mercaptoethanol, 20% glycerol). Nuclei from 1/5 of each preparation were treated with four ul of RNAse A, 10 ug/ul, (Qiagen) and used for Micrococcal Nuclease (Takara) digestion for 6 minutes (final concentration 0.2 U/ul) in digestion buffer (16 mM Tris-Cl, pH 7.6, 50 mM NaCl, 2.5 mM CaCl_2_, 0.01 mM PMSF and EDTA-free protease inhibitor cocktail (Roche) and stopped with 10 mM EDTA. Mononucleosomes were released by treating nuclei with 0.1% Triton-X for 1–2 hours in the cold, and then pelleting the debris by centrifuging at 3500 rpm for 3 min (Eppendorf, 5415R). 500 ul of supernatant was applied to 50 ul of Dynabeads Protein A (Invitrogen) that were preincubated with 2.5 ug of the appropriate antibody (#1220, monoclonal anti-H3K9m2 antibody, Abcam; #1791, polyclonal anti-H3 antibody, Abcam) in buffer (20 mM Tris-Cl, pH 7.6, 50 mM NaCl, 5 mM EDTA and 0.1% Triton) and incubated overnight. Beads were washed (10 min incubation in the cold) with 500 ul of the following buffers: 50 mM Tris-Cl pH 7.6, 10 mM EDTA, 0.1 mM PMSF, protease inhibitor cocktail (Roche) with changing concentration of NaCl to 50 mM, 100 mM and 150 mM, subsequently. Final wash was done in TE buffer, without incubation and immunocomplexes were eluted with 500 ul of 0.1% SDS and 0.1 M NaHCO_3_ at 65°C for 10 min. DNA was then purified using conventional phenol-chlorophorm extraction and ethanol/salt precipitation. DNA amplification was performed using the GenomePlex® Whole Genome Amplification Kit (Sigma). Four amplification reactions were performed in parallel for each sample to minimize spurious amplification artifacts and purified using QlAquick spin columns (Qiagen) and the products were combined. 4–6 ug of DNA was obtained and Roche Nimblegen performed array hybridization, washing and scanning. For the ChIP with crosslinked tissue, a previously described protocol was used [45].

### Microarray design and data analysis

Together with Roche Nimblegen, we designed a forward strand Arabidopsis whole-genome array which contained ∼1.98 million 60-nt oligonucleotide features. Of the ∼1.98 million features, a total number of ∼1.87 million features matched a unique position in the genome. Three biological replicas were performed for the MNase digested ChIP samples. Each probe was assigned a log of the ratio between H3K9m2(Cy5) and H3(Cy3) signal. Z-scores were calculated for each probe by subtracting the mean and dividing by the standard deviation for the entire array. The Z-scores for each probe were averaged among three replicas, and the correlation between replicates was high (Pearson correlation coefficient between 0.85 and 0.9).

Based on the distribution of Z-scores (Figure S2), we defined a positive probe as having a score higher than 0.2, which gave a false positive rate in the chloroplast genome of 1.19%. In order to eliminate false positive signals from single probes, we defined a set of “methylated regions” by combining adjacent probes with scores higher than 0.2, allowing a maximal gap of 120 base pairs, and minimum run of 60 base pairs by using Integrated Genome Browser (Affymetrix). This setting allows detection of regions with a size of a mononucleosome (147 bp), but eliminates signal from single isolated probes (60 bp).

TAIR7 release of Arabidopsis genome was used for annotations of genes and transposon/pseudogenes. DNA methylation data is described in [32]. Positions of tandem, inverted and interspersed repeats, as well as expression data for gene are based on [39]. siRNA cluster data is based on the study by [51]. The data is available at http://epigenomics.mcdb.ucla.edu/H3K9m2/. Two tracks ‘H3K9m2MD:ZLrToH3’ and ‘H3K9m2CL:ZLrToH3’ represent data from native and crosslinked ChIP experiments, respectively. The default display range of z-scores is 0.0 to 2.0 with a horizontal line at 1.0. Microarray data reported in the manuscript is deposited to GEO at NCBI, series accession number GSE12383 (in accordance with MIAME guidelines).

## Supporting Information

Figure S1 MNase digestion conditions and specificity of anti-H3K9m2 antibody. (a) Nuclear DNA digested with MNase for various periods of time. Six-minute digestion was used for ChIP assays. (b) Western blot analysis of interaction of anti-H3K9me2 antibody with different methylated peptides.
(2.93 MB TIF)

Figure S2 Distribution of H3K9m2 levels (Z-scores) for probes corresponding to each of the five Arabidopsis nuclear chromosomes and chloroplast. Histogram shows the distribution of the numbers of probes relative to their Z-score. Black line (0.2) indicates the cut off value used for determining H3K9m2 positive probes.
(2.06 MB TIF)

Figure S3 Examples of H3K9m2 patterns observed using two different techniques for chromatin immunoprecipitation, micrococcal nuclease digested chromatin (MNase) or crosslinked and sonicated chromatin (Crosslink). Each gray bar corresponds to the z-score of an individual probe. Genes are in green and transposons are in red.
(3.10 MB TIF)

Figure S4 Correlation of H3K9m2 methylation with CHG DNA methylation. Examples show the tight association between H3K9m2 positive regions (represented by grey signal), and CHG methylation as determined by whole genome bisulfite sequencing. Bright blue rectangles represent CHG methylation and green and red rectangles represent CG and CHH methylation respectively.
(2.65 MB TIF)

Figure S5 Genes are devoid of H3K9m2 and CHG methylation and have high frequency of CG methylation. Examples show the lack of association between H3K9m2 positive regions (represented by grey signal), and CG only methylated regions associated with the transcribed regions of genes, as determined by whole genome bisulfite sequencing. Green rectangles represent CG methylation and blue and red rectangles represent CHG and CHH methylation respectively.
(3.24 MB TIF)

Figure S6 Chromosome-wide coordinates for pericentromeric (dark) and euchromatic arm regions in megabases. Pericentromeric regions were assigned based on the distribution of repetitive elements, genes and DNA methylation across chromosomes.
(0.33 MB TIF)

Figure S7 Distribution of Z-scores of probes found in pericentromeric regions or euchromatic arms of the chromosomes. Data was generated by conventional (crosslinked) ChIP.
(0.39 MB TIF)

Table S1 Regions previously annotated as H3K9m2 methylated and their overlap with H3K9m2 methylation found in current study.
(0.16 MB XLS)
