# Supplementary figures and images for: Genome-Wide Association of Histone H3 Lysine Nine Methylation with CHG DNA Methylation in Arabidopsis thaliana
Source: PLoS One. 2008 Sep 8;3(9):e3156. doi: 10.1371/journal.pone.0003156 (PMC2522283; doi:10.1371/journal.pone.0003156)

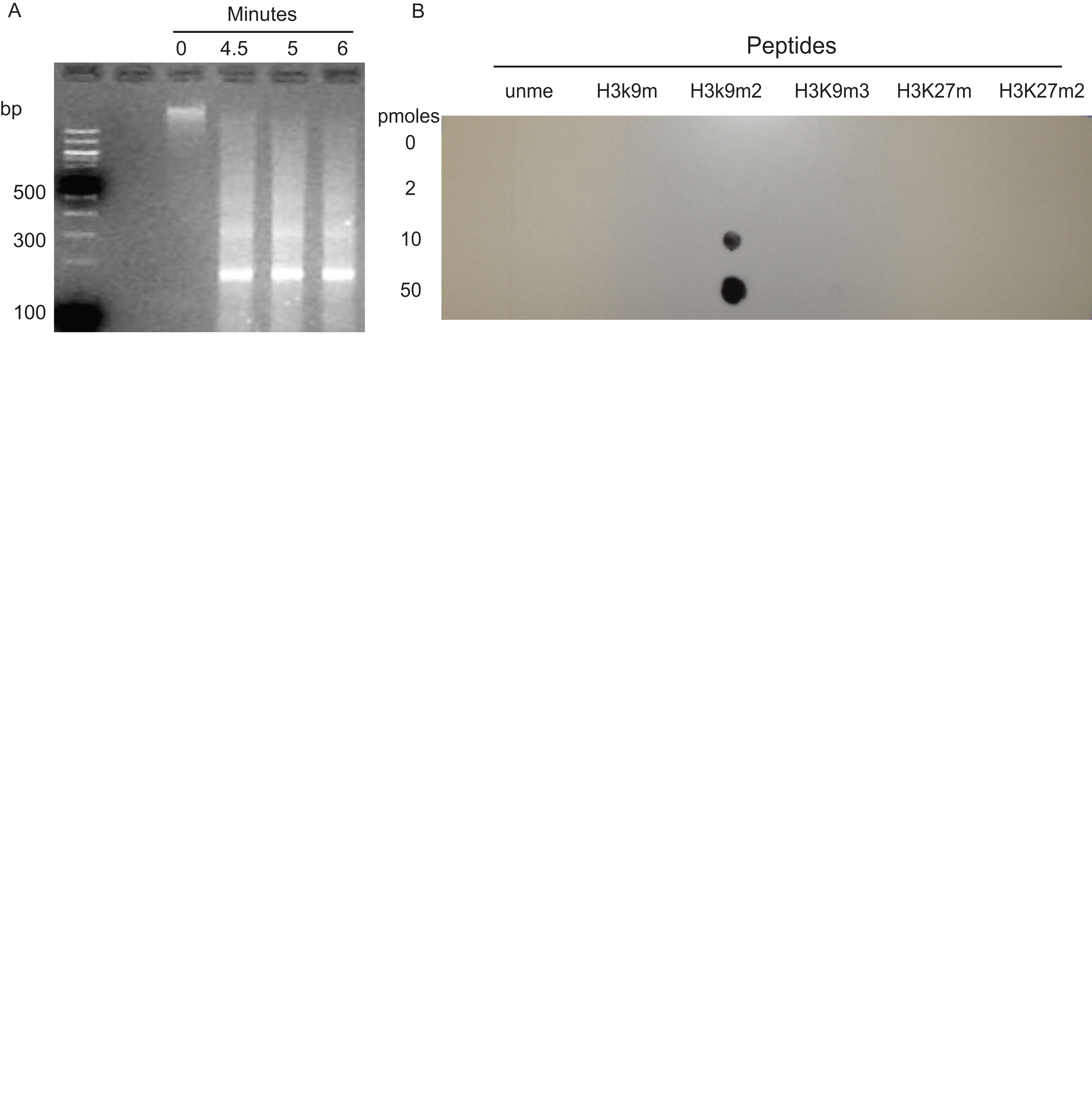

Supplement: Figure S1 — MNase digestion conditions and specificity of anti-H3K9m2 antibody. (a) Nuclear DNA digested with MNase for various periods of time. Six-minute digestion was used for ChIP assays. (b) Western blot analysis of interaction of anti-H3K9me2 antibody with different methylated peptides. (2.93 MB TIF) [file pone.0003156.s001.tif]

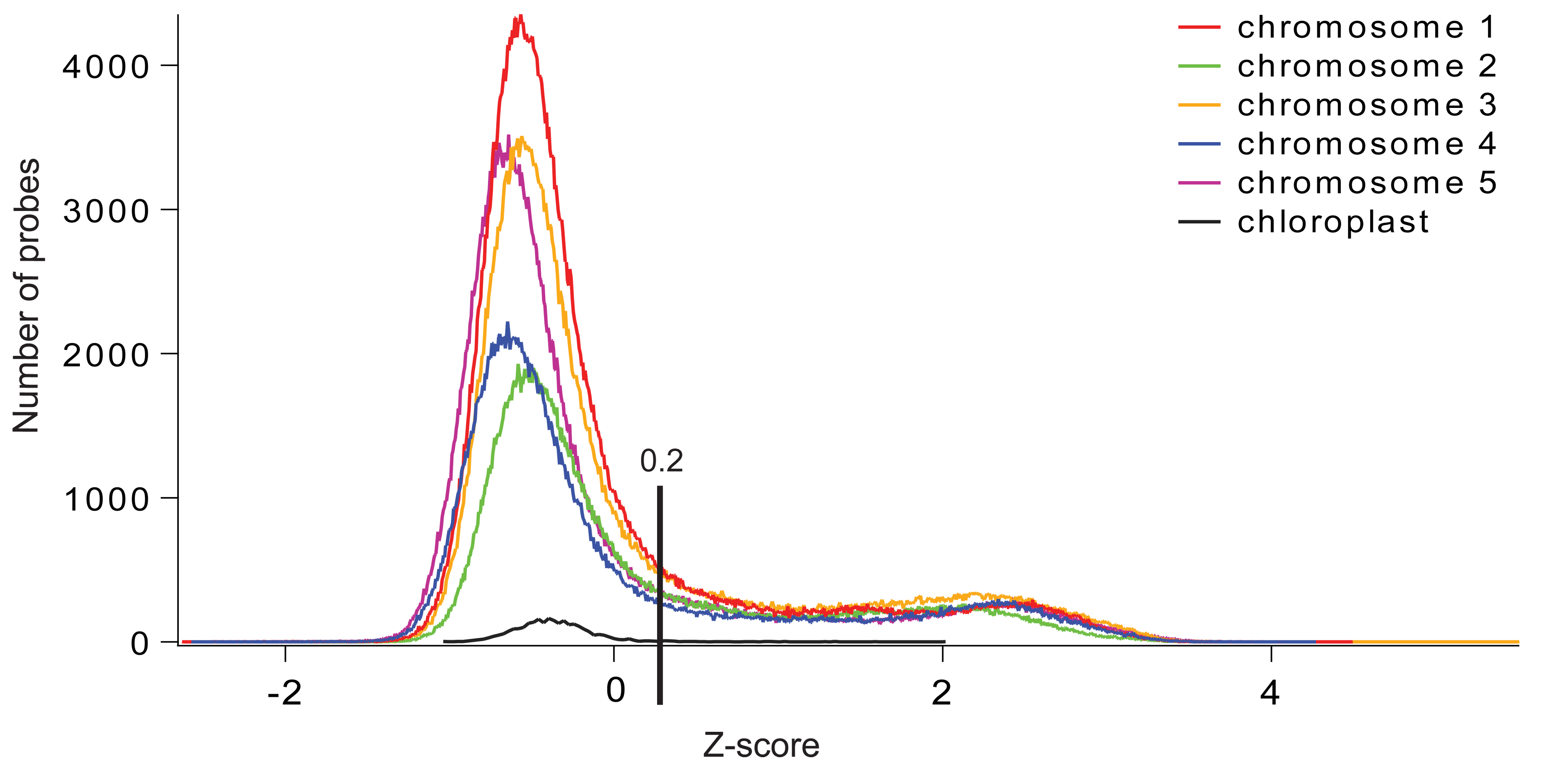

Supplement: Figure S2 — Distribution of H3K9m2 levels (Z-scores) for probes corresponding to each of the five Arabidopsis nuclear chromosomes and chloroplast. Histogram shows the distribution of the numbers of probes relative to their Z-score. Black line (0.2) indicates the cut off value used for determining H3K9m2 positive probes. (2.06 MB TIF) [file pone.0003156.s002.tif]

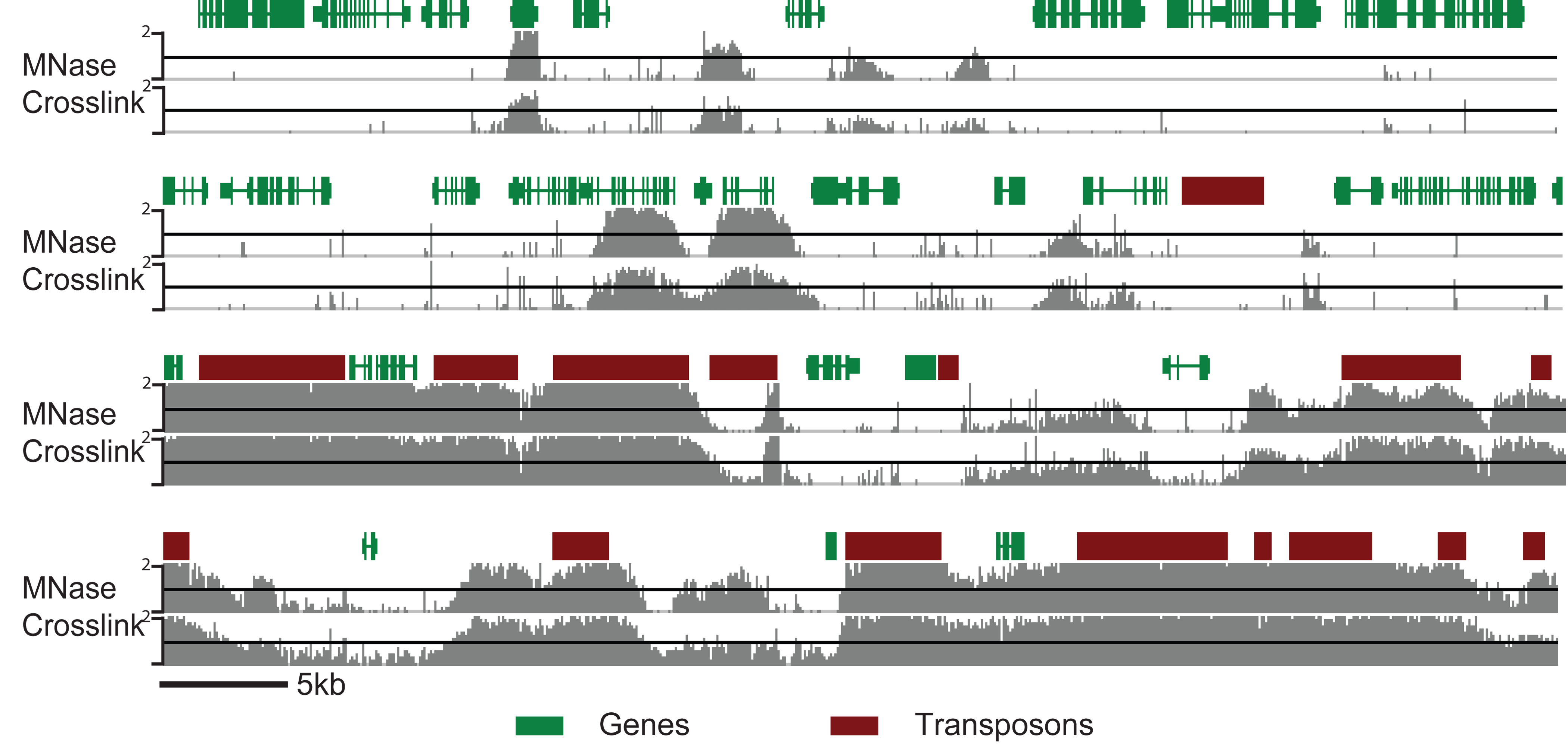

Supplement: Figure S3 — Examples of H3K9m2 patterns observed using two different techniques for chromatin immunoprecipitation, micrococcal nuclease digested chromatin (MNase) or crosslinked and sonicated chromatin (Crosslink). Each gray bar corresponds to the z-score of an individual probe. Genes are in green and transposons are in red. (3.10 MB TIF) [file pone.0003156.s003.tif]

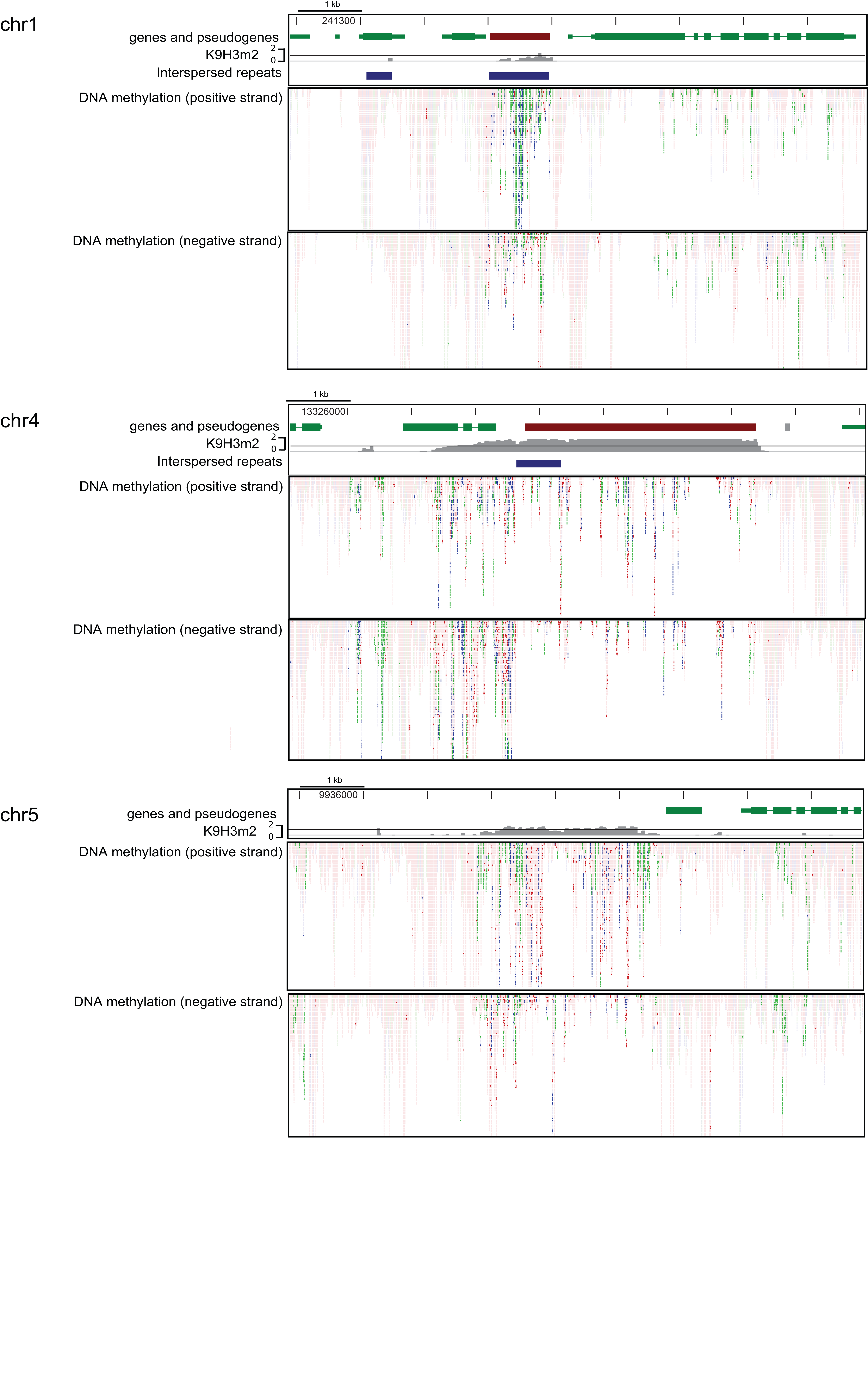

Supplement: Figure S4 — Correlation of H3K9m2 methylation with CHG DNA methylation. Examples show the tight association between H3K9m2 positive regions (represented by grey signal), and CHG methylation as determined by whole genome bisulfite sequencing. Bright blue rectangles represent CHG methylation and green and red rectangles represent CG and CHH methylation respectively. (2.65 MB TIF) [file pone.0003156.s004.tif]

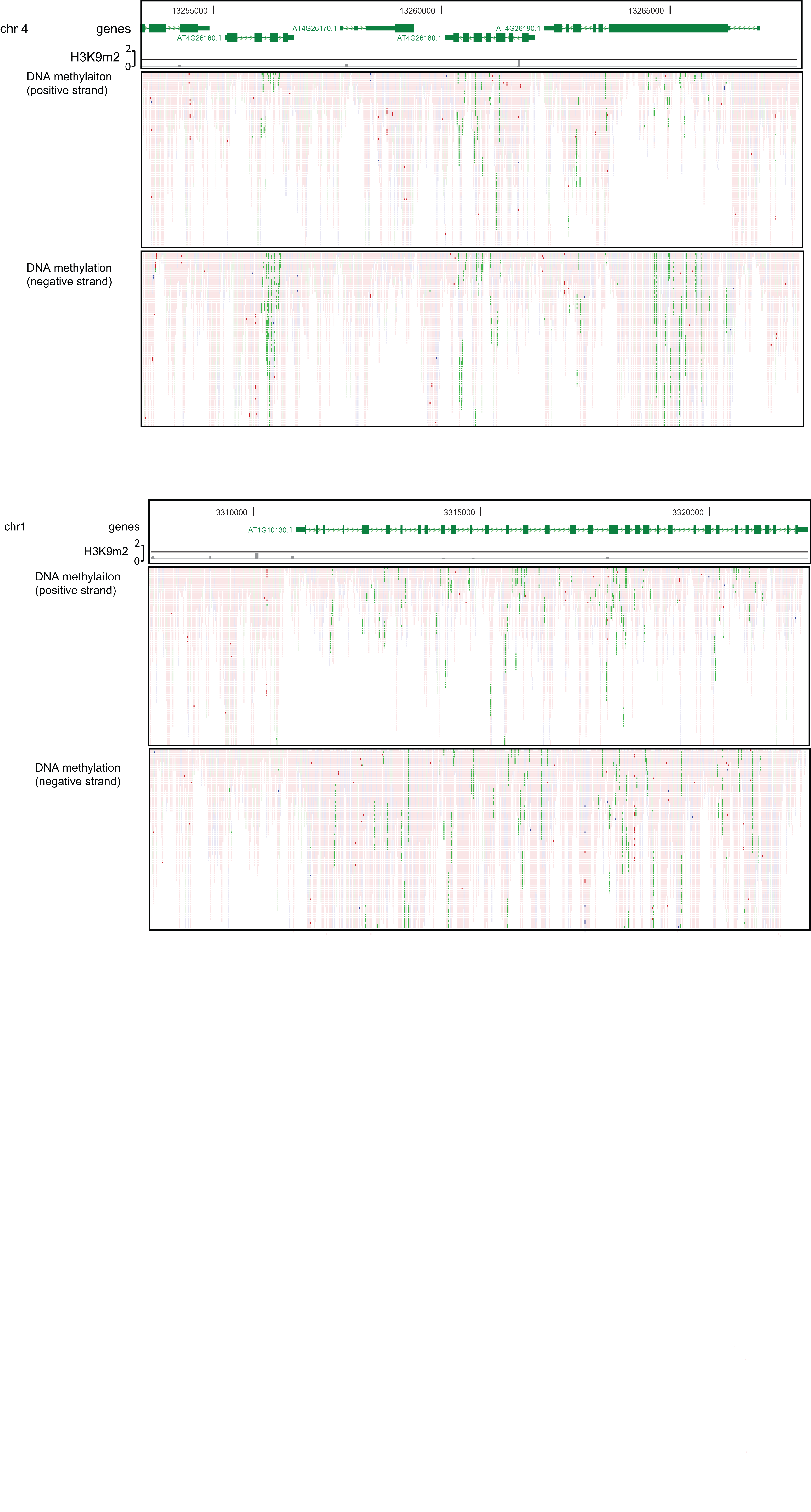

Supplement: Figure S5 — Genes are devoid of H3K9m2 and CHG methylation and have high frequency of CG methylation. Examples show the lack of association between H3K9m2 positive regions (represented by grey signal), and CG only methylated regions associated with the transcribed regions of genes, as determined by whole genome bisulfite sequencing. Green rectangles represent CG methylation and blue and red rectangles represent CHG and CHH methylation respectively. (3.24 MB TIF) [file pone.0003156.s005.tif]

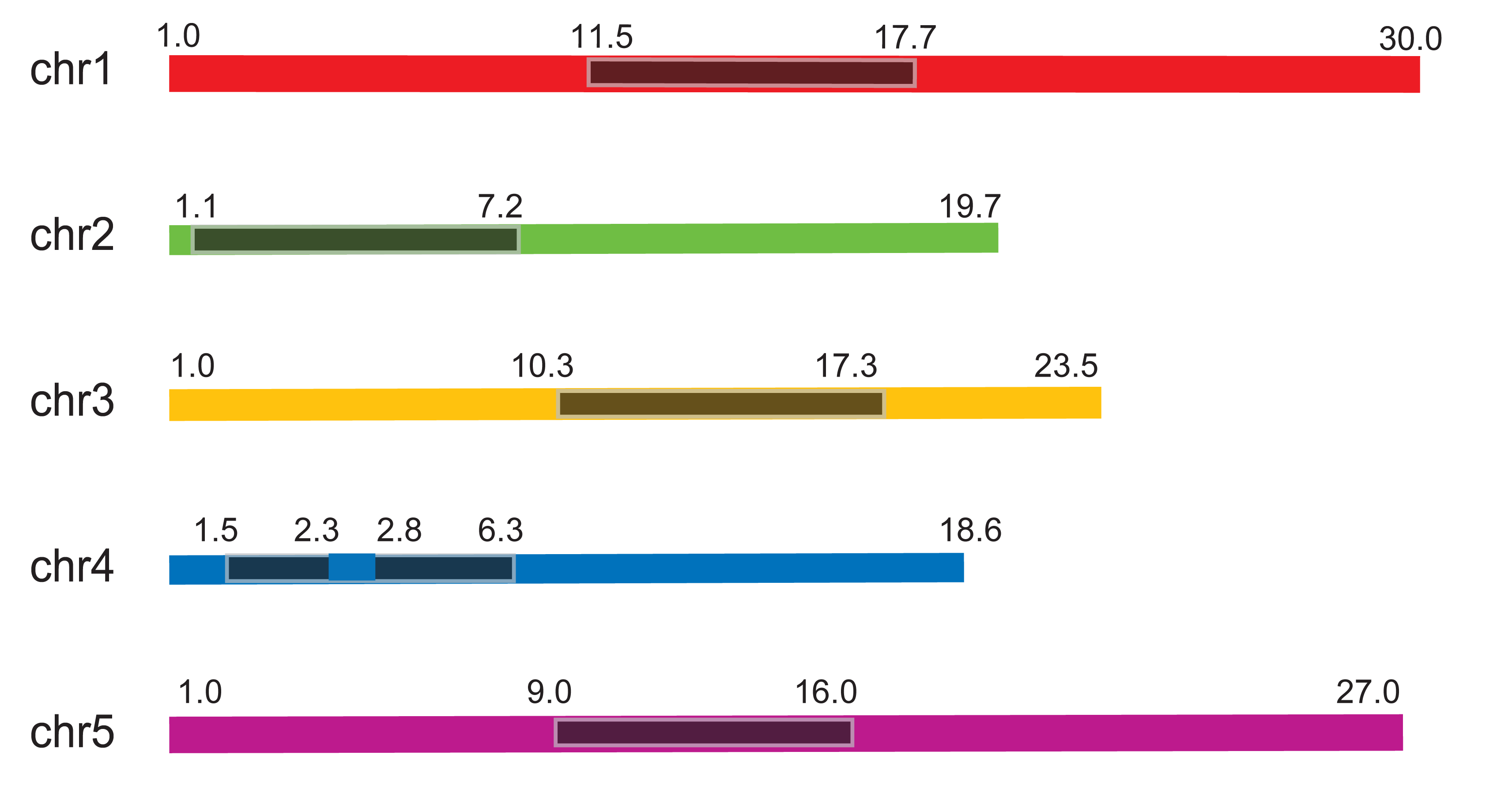

Supplement: Figure S6 — Chromosome-wide coordinates for pericentromeric (dark) and euchromatic arm regions in megabases. Pericentromeric regions were assigned based on the distribution of repetitive elements, genes and DNA methylation across chromosomes. (0.33 MB TIF) [file pone.0003156.s006.tif]

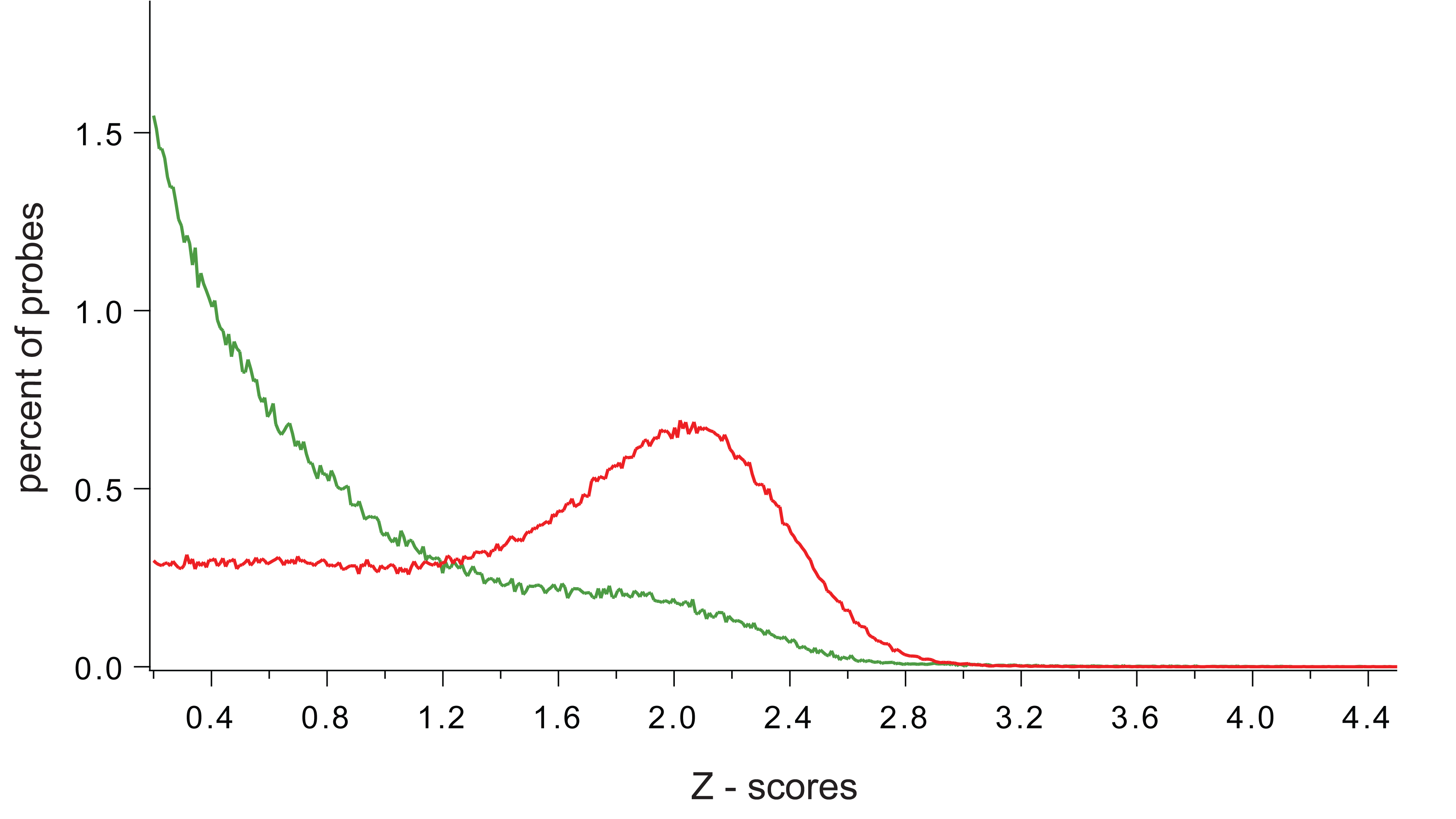

Supplement: Figure S7 — Distribution of Z-scores of probes found in pericentromeric regions or euchromatic arms of the chromosomes. Data was generated by conventional (crosslinked) ChIP. (0.39 MB TIF) [file pone.0003156.s007.tif]
